# Genotyping-By-Sequencing (GBS) Detects Genetic Structure and Confirms Behavioral QTL in Tame and Aggressive Foxes (*Vulpes vulpes*)

**DOI:** 10.1371/journal.pone.0127013

**Published:** 2015-06-10

**Authors:** Jennifer L. Johnson, Helena Wittgenstein, Sharon E. Mitchell, Katie E. Hyma, Svetlana V. Temnykh, Anastasiya V. Kharlamova, Rimma G. Gulevich, Anastasiya V. Vladimirova, Hiu Wa Flora Fong, Gregory M. Acland, Lyudmila N. Trut, Anna V. Kukekova

**Affiliations:** 1 Department of Animal Sciences, College of ACES, University of Illinois at Urbana-Champaign, Urbana, IL, 61801, United States of America; 2 Baker Institute for Animal Health, Cornell University, College of Veterinary Medicine, Ithaca, NY, 14853, United States of America; 3 Institute of Biotechnology, Genomic Diversity Facility, Cornell University, Ithaca, NY, 14853, United States of America; 4 Institute of Cytology and Genetics of the Russian Academy of Sciences, Novosibirsk, 630090, Russia; Texas A&M University, UNITED STATES

## Abstract

The silver fox (*Vulpes vulpes*) offers a novel model for studying the genetics of social behavior and animal domestication. Selection of foxes, separately, for tame and for aggressive behavior has yielded two strains with markedly different, genetically determined, behavioral phenotypes. Tame strain foxes are eager to establish human contact while foxes from the aggressive strain are aggressive and difficult to handle. These strains have been maintained as separate outbred lines for over 40 generations but their genetic structure has not been previously investigated. We applied a genotyping-by-sequencing (GBS) approach to provide insights into the genetic composition of these fox populations. Sequence analysis of *Eco*T22I genomic libraries of tame and aggressive foxes identified 48,294 high quality SNPs. Population structure analysis revealed genetic divergence between the two strains and more diversity in the aggressive strain than in the tame one. Significant differences in allele frequency between the strains were identified for 68 SNPs. Three of these SNPs were located on fox chromosome 14 within an interval of a previously identified behavioral QTL, further supporting the importance of this region for behavior. The GBS SNP data confirmed that significant genetic diversity has been preserved in both fox populations despite many years of selective breeding. Analysis of SNP allele frequencies in the two populations identified several regions of genetic divergence between the tame and aggressive foxes, some of which may represent targets of selection for behavior. The GBS protocol used in this study significantly expanded genomic resources for the fox, and can be adapted for SNP discovery and genotyping in other canid species.

## Introduction

The red fox (*Vulpes vulpes*) and the gray wolf (*Canis lupus*), both members of the Canidae family, diverged from a common ancestor approximately 10 million years ago [1, 2]. The domestic dog (*Canis lupus familiaris*) is a recent descendant of the modern gray wolf ancestor and the only historically domesticated canid. In 1959, work began at the Institute of Cytology and Genetics (ICG, Novosibirsk, Russia) to domesticate the silver fox, a coat color variant of the red fox [3–7]. Here, a tame fox strain was produced from conventionally bred farm foxes by first eliminating fearful and aggressive animals from the breeding population and then selecting for friendly behavior to humans [5, 8–10]. The rapid response to selection for behavior during the first ten generations [5, 6, 9, 10] strongly suggests that selection was acting on preexisting genetic variation that was present in the founder population. The effort to minimize inbreeding in the fox population during the entire the breeding program [11–14] has allowed continued, and ongoing selection for behavior (for review [5, 8–10]). The tame strain foxes are eager to establish human contact and demonstrate friendly, playful behavior towards humans, paralleling the sociability of dog puppies. The tame population was maintained as a closed line and currently comprises approximately 300 breeding animals.

Since commercial farms usually eliminate animals with an excessively aggressive response to humans, a parallel breeding program was begun in 1970 to preserve this behavior for research [6, 10]. Selection of breeding animals was based on fox awareness (critical distance between the experimenter and the caged animal when the fox demonstrates a hostile behavior) and the intensity of the fox’s aggressive response. Foxes from the aggressive strain avoid interaction with humans and are aggressive when approached. The aggressive population was largely maintained as a closed line but an introgression of farm-bred foxes that had not been systematically selected for behavior was made in the 1990s. The current population of aggressive foxes comprises approximately 150 breeding animals [5, 8–10].

The genetic basis of tame and aggressive behavioral phenotypes has been clearly demonstrated in multiple experiments [6, 8, 12]. Using the current genomic resources for the dog (i.e., microsatellite loci) the fox meiotic linkage map has been constructed [11–13] and used for the genetic mapping of fox behavioral phenotypes [12]. Although the dog and fox have very different karyotypes, the dog having 78 chromosomes and the fox 34 chromosomes and 0–8 B chromosomes, the conservation of synteny between the dog and fox genomes is well established [11, 13, 15–17]. Alignment of the fox meiotic linkage map against the dog genome has been used to identify positions of fox behavioral loci in the dog genome and to predict the locations of the gene orthologs on the fox chromosomes [18].

Genotyping-by-sequencing (GBS), like some other current technologies [19–21], uses restriction enzymes (REs) to reduce genome complexity and next-generation sequencing for simultaneous SNP discovery and genotyping [22]. Originally designed for SNP genotyping in plant species with sequenced genomes, the protocol can be adapted to a wide variety of species, including animals [23], either with or without reference sequence information [24]. GBS, however, had not been previously used for genotyping canids. In the current study, we optimized the GBS protocol for the silver fox and genotyped a subset of individuals from the tame and aggressive strains. The resulting SNP data were used to analyze the genetic structure of the fox strains and to provide information critically needed for designing genetic mapping studies in these populations.

In a previous study, quantitative trait loci (QTL) analysis of fox experimental pedigrees identified several regions in the fox genome implicated in behavior [12] (Nelson et al., in preparation). Because the fox meiotic linkage map did not cover distal regions of several fox chromosomes, these regions have not been included in the QTL analysis. In this study, positions of the regions which showed allele frequency differences between the tame and aggressive populations were compared with the positions of previously identified behavioral QTLs. The region on the distal part of the fox chromosome 3 which showed significant allele frequency difference but was not included in the previous QTL analysis was evaluated in a greater detail to asses the effect of this region on behavior.

Behavioral differences between tame and aggressive fox strains have been maintained for many decades and generations [5, 8–10]. Importantly, the intense selection for behavior in these strains has been combined with a strenuous effort to avoid inbreeding [5, 6, 8–10]. The fox strains may provide a more robust model for identification of targets of selection for behavioral traits than the domestic dog where selection was acting on different traits including appearance and morphology. Genetic analysis of these fox populations should facilitate identification of loci and genes involved in regulation of behavioral traits in canids.

## Materials and Methods

### Samples

Blood samples were collected from foxes maintained at the experimental farm of the Institute of Cytology and Genetics (ICG) in Novosibirsk, Russia. All animal procedures at the ICG complied with standards for humane care and use of laboratory animals by foreign institutions. The study was approved by the Institutional Animal Care and Use Committee (IACUC) of the University of Illinois at Urbana-Champaign. DNA was extracted from blood using Qiagen Maxi Blood kits, as per the manufacturer’s instructions (Qiagen, Valencia, CA).

Three sets of DNA samples we used in this study:

1. Genotyping-by-sequencing (GBS) set. We used samples from 40 individuals, 20 each from the tame and aggressive populations, for the genotyping-by-sequencing (GBS) experiment. Each population sample included equal numbers of males and females, and individuals were not closely related (i.e. did not share parents or, in most cases, grandparents).
2. Validation set. The second sample set was selected to validate the differences in allele frequency of SNPs from the distal part of fox chromosome 3 (VVU3) which were observed between the tame and aggressive populations using GBS data. The validation set included 45 aggressive, 46 tame, and 92 conventional farm-bred foxes (foxes which were not deliberately selected for behavior). Again, animals were, generally, not closely related and animals from both genders were equally represented.
3. Genetic mapping set. Fox F2 pedigrees including parents, grandparents, and 536 offspring were used for construction of a meiotic linkage map of VVU3 and quantitative trait loci (QTL) analysis. The F2 pedigrees were developed in a previous study [12, 25] by crossbreeding tame and aggressive foxes and then breeding F1 individuals to each other. All F1 pedigrees were produced in reciprocal manner with respect to parental gender and population of origin. Behavior of F2 individuals was tested, videotaped and scored from video records with a set of 98 traits [7, 12, 25]. Principal component (PC) analysis was used to define main axes of fox behavior as previously described [12]. Both PC defined phenotypes and scores for individual behavioral traits were used for QTL mapping [12, 25] (Nelson et al., in preparation).

### Restriction enzyme (RE) selection and GBS optimization

To determine the best restriction enzyme (RE) system for reducing the complexity of the fox genome, DNA (100 ng) from a single individual was digested, separately, with several REs (*Ape*KI, *Eco*T22I, *Pst*I, and *Eco*T22I/*Ps*tI double digest) according to the enzyme manufacturer’s protocol (New England Biolabs, Ipswich, MA). Samples were digested at 37°C for 2 hours and then, incubated at 80°C for 20 minutes to inactivate REs. Digested DNAs were ligated to adapters as previously described [22]. Samples were then purified (QIAquick PCR Purification Kit; Qiagen, Valencia, CA) and 2 μl of each library was amplified in a 50 μl volume containing 1 x *Taq* master mix (New England Biolabs, Ipswich, MA) and 25 pmol of each of two primers containing complementary sequences to ligated adapters and Illumina solid-phase oligonucleotides bound to the flowcell lane surface. PCR was performed following the previously described protocol [22]. Amplified libraries were purified again, as above, and fragments were visualized using the Experion (Bio-Rad, Hercules, CA) (S1 Fig). DNA profiles for *Ape*KI and *Eco*T22I enzymes showed enrichment for fragments of desirable sizes (S1 Fig) and did not contain repeat-associated peaks, therefore libraries were made with both of these enzymes.

### GBS library preparation and DNA sequencing

The first run of sequencing was done with separate libraries made with *Ape*KI or *Eco*T22I enzymes [22]. Forty-seven fox DNA samples comprising 20 tame, 20 aggressive, and seven duplicated individuals were genotyped. The DNA samples (100 ng) were digested, separately, with *Ape*KI or *Eco*T22I at 37°C for 2 hours, and each sample was ligated to equal amounts of a different barcode-containing adapter and the same common adapter. The 48 barcode sequences used for *Eco*T22I GBS library construction (a negative control was also included) are listed in S1 Table. The barcode sequences used for *Ape*KI GBS library construction were published previously [22]. Individual ligation reactions (5 μL each) were pooled and DNA was purified (QIAquick PCR Purification Kit; Qiagen, Valencia, CA). A sample from the pooled library (10 μL) was amplified by PCR (50 μL total volume) containing 1 x *Taq* master mix (New England Biolabs, Ipswich, MA) and 12.5 pmol of each PCR primer (see S1 Table; [22]). PCR products were purified as above and quantified using the Nanodrop 2000 (Thermo Scientific, Wilmington, DE). First, the *Ape*KI or *Eco*T22I GBS libraries were sequenced on a HiSeq 2000 (Illumina Inc., San Diego, CA) in the same sequencing flowcell lane (single-end sequencing with 100 bp reads), with approximately half of the sequencing coming from each.

The average number of reads for the *Ape*KI library was 1,482,234, with high variation in read numbers among samples (SD± 902,649; median: 1,569,610). Nine animals received so little sequence that they were judged as failed. This was likely due to technical errors during the library preparation. Due to uneven results we did not use sequencing data generated for the *Ape*KI library in the following analysis.

The average number of reads per sample for the *Eco*T22I library was 1,181,370 (SD +/-314,990; median: 1,066,520) and no samples failed. Because the *Eco*T22I library produced good results in the first run we sequenced already created *Eco*T22I library on an additional flowcell lane to obtain deeper sequencing data for this library. This strategy produced coverage depths equivalent to 32-plex sequencing.

### SNP discovery

SNPs were called from raw DNA sequences using the GBS pipeline as implemented in TASSEL 3.0.166 [26]. Raw sequences were converted to tag counts using the FastqToTagCountPlugin (options:-c 1, -s 300000000), where-c 1 is the minimum taxa count within a qseq file for a tag to be output; default: 1;-s 300000000 is the maximum number of tags the TBT (tags-by-taxa) can hold while merging (default: 200000000). The tag counts were merged using the MergeMultipleTagCountPlugin (options:-c 3). A fastq file with unique 64 bp sequence tags was created using the TagCountToFastqPlugin (options:-c 1), and aligned to the dog genome (//hgdownload.soe.ucsc.edu/goldenPath /canFam3/bigZips/canFam3.fa.gz accessed on 31 March 2014 with md5sum 07084e3a9843991825a67891d23e3735). Dog chromosomes were renamed for compatibility with the GBS pipeline (leading ‘chr’ notations were removed, and X and M chromosomes were converted to 39 and 40). The modified dog genome was indexed for use with BWA version 0.7.8-r455 [27] and the fox tags were aligned to it with BWA aln/samse using the default parameters. TBT files were created using the FastqToTBTPlugin (options:—c 1, -y) and merged using the MergeTagsByTaxaFiles plugin (options:-s 300000000). SNPs were called with the tbt2vcfPlugin (options:-ak 3, -mnLCov 0, -mnMAF 0), where-ak 3 is the maximum number of alleles that are kept for each marker across the population; default: 3;-mnLCov 0 is the minimum locus coverage (proportion of taxa with a genotype; default: 0.0;-mnMAF 0 is the minimum minor allele frequency; default: 0.0 = no filter.

Duplicate SNPs were merged with the MergeDuplicateSNP_vcf_Plugin (options:-ak 3). For VCF files, likelihood scores were calculated according to Etter et al., 2011, formula 3.8 [28]. The most likely genotype was assigned, and a genotype quality (GQ) score was calculated according to GATK:

“GQ: The Genotype Quality, or Phred-scaled confidence that the true genotype is the one provided in GT. In the diploid case, if GT is 0/1, then GQ is really L(0/1) / (L(0/0) + L(0/1) + L(1/1)), where L is the likelihood that the sample is 0/0, 0/1/, or 1/1 under the model built for the NGS dataset. The GQ is simply the second most likely PL—the most likely PL. Because the most likely PL is always 0, GQ = second highest PL—0. If the second most likely PL is greater than 99, we still assign a GQ of 99, so the highest value of GQ is 99.”

(http://gatkforums.broadinstitute.org/discussion/1268/how-should-i-interpret-vcf-files-produced-by-the-gatk accessed on 21 November 2014)

TASSEL 4.3.7 was used to merge 7 replicated samples using the plugin MergeIdenticalTaxaPlugin (options:-hetFreq 0.8, -maxAlleleVCF 3) [26].

### SNP filtering

Two sets of SNPs, filtered and stringently filtered sets, were produced using VCFtools [29]. Filtering was initially performed using the following parameters: (1) the SNP was called at least 50% of individuals (--max-missing 0.5), (2) minor allele frequency (MAF) was > 2.5% (--—maf 0.025), (3) only two alleles were present (--min-alleles 2--max-alleles 2), (4) average read depth < 150 (--max-meanDP 150), (5) < 60% heterozygous individuals for autosomal SNPs and the X chromosome pseudoautosomal region (PAR) (0–6.65Mb) [30], or > 30% heterozygous individuals for the remainder of the X chromosome. More stringent filtering was performed by eliminating loci with quality score < 98 (considers sequence depth per locus) (--minGQ 98) and SNPs with missing data (--max-missing 1)

### SNP evaluation

Consistency of SNP genotype calling was evaluated by comparing the two replicates from each of the seven duplicated DNA samples. Both filtered and stringently filtered SNP data sets were created for the unmerged data (where replicates of the duplicated samples were treated as separate samples), in the same manner as described above for the merged data set. In the filtered set, two types of discordance between the replicates were observed: 1) when one sample in a pair did not receive a call but the other did (missing data) and 2) SNPs for which both samples in the pair received a genotype call but the genotypes differ between the replicates. In the stringently filtered set, only second parameter was present because all SNPs in this data set had genotyping calls for all samples. The percent of concordant genotypes was calculated for each of the duplicated individuals in both the filtered and stringently filtered sets. The percent of the discordant genotypes due to missing data was also calculated for each duplicated individual but only in the filtered set.

The depth of the read coverage for the concordant and discordant SNPs was determined using the unmerged, filtered set of SNPs. The discordant genotypes due to missing data were not included in this analysis, only those SNPs that have genotypes for both replicates of the duplicated individuals were used. The mean and standard deviation of the depth was calculated for both concordant and discordant SNPs in this data set. The average heterozygosity of SNPs located on the X chromosome outside of PAR (0–6.65Mb) [30] was calculated. This was computed for males and females in each population separately by determining the percent of individuals of the same gender with heterozygous genotypes for each SNP, summing the values and dividing by the number of SNPs with data.

### Estimating SNP positions on fox chromosomes

The location of GBS SNPs on the fox chromosomes was estimated by alignment of the fox meiotic linkage map against the dog genome as previously described [11, 13]. Briefly, dog chromosomes were divided into corresponding “fox segments” based on the location of known mapped SSR markers. When dog chromosomes were split into syntenic regions located on two fox chromosomes, the length of the entire region between known dog markers was included on both of the corresponding fox chromosomes. This was done to prevent markers that might be far apart in the fox genome from appearing to be close together on the estimated fox map. SNPs that mapped to unassigned portions of the dog genome (regions with unknown synteny and “Un” chromosomes) were not mapped to fox positions.

### Population structure analysis, estimation of LD and effective population size

The stringently filtered SNP set (8,437 SNPs) was used for population structure analysis using Principal Component (PC) analysis and the Bayesian inference program STRUCTURE [31]. PC analysis was performed using PLINK2 (https://www.cog-genomics.org/plink2) [32]. Clustering analysis was performed using STRUCTURE v.2.3.4 [31, 33] at 100,000 iterations of the Gibbs sampler after a burn-in of 100,000 iterations. Each run was repeated eight times at each value of K using correlated allele frequency model with admixture model with default settings. The runs were completed for K from 2 to 5 without the population information.

A subset of stringently filtered SNPs, whose positions were extrapolated on fox chromosomes (8,405 SNPs), was used to estimate linkage disequilibrium (LD) decay in both tame and aggressive populations. Calculations of the squared correlation of the alleles at two loci (r^2^) were done in PLINK2 [32]. Distances between SNPs were estimated using the inferred SNP positions on fox chromosomes and were based on the corresponding physical distances in the dog genome. Average r^2^ between adjacent SNPs was computed by grouping SNPs by pairwise estimated physical distances into 14 bins ranging from 1–1000 bp to 100–210 Mb (size of the largest approximated fox chromosome is 210 Mb) (S2 Table). LD was also examined between SNPs located on different chromosomes (i.e., r^2^ was computed for all pairs of SNPs in which both SNPs were located on separate fox chromosomes).

The set of stringently filtered mapped SNPs was also used to calculate effective population size (Ne) using the LD method of Waples and Do [34] as implemented in NeEstimator [35].

### Estimation of population parameters

The filtered SNP set (48,294 SNPs) was used to calculate population parameters. Observed heterozygosity was computed as the percent of heterozygous individuals (calculated separately for each population) among the total number of individuals that received genotype calls for that SNP. The average observed heterozygosity of the population was calculated as a sum of the SNP heterozygosity in that population divided by the total number of SNPs identified in the population. The mean, median and standard deviation of the minor allele frequency (MAF) were calculated separately for the tame and aggressive populations. Expected heterozygosity was calculated for each SNP by formula 2.16b from Hedrick, 4th Edition, page 93 [36]. The obtained value was divided by the number of individuals in the population with data (N) to calculate the proportion of individuals that would be expected to be heterozygous given the allele frequencies seen (the formula 2.16b calculates the expected number of individuals expected to be heterozygous). To calculate the mean expected heterozygosity, the expected heterozygosity values for each SNP within each population were summed and the obtained numbers were divided by the total number of SNPs.

### Scan for SNP allele frequency differences between the fox populations

To identify regions in the fox genome where the two populations differed in allele frequency, 48,042 SNPs from the filtered set that mapped to the fox genome were compared between the tame and aggressive samples using the (--assoc--adjust) option in PLINK2 [32] with Bonferroni correction.

In addition to the above analysis, we also computed fixation index (F_ST_) using VCFtools [29]. The value for “weighted_F_ST_” is reported. The F_ST_ was calculated in windows of 1 Mb with the overlap of 500 Kb using the Weir and Cockerham estimator [37] (--weir-fst-pop aggr--weir-fst-pop tame--fst-window-size 1000000--fst-window-step 500000)

### Confirmation of allele frequencies and construction of the fox chromosome 3 (VVU3) map

To confirm and further characterize a region of genetic divergence between tame and aggressive populations on fox chromosome 3 (VVU3) and to extend the existing linkage map for this chromosome, four new fox markers (three SSRs and one indel) were developed. The indel was identified through sequencing fox amplicons produced using dog derived primers; two of the SSR markers corresponded to microsatellites identified in the dog genome, and one SSR was selected from the dog meiotic linkage map [38]. Primers were designed with Primer3 [39] using the dog genome sequence. Fluorescent primers (S3 Table) were genotyped following the protocol described in Kukekova et al., 2007 [11]. Three SSR markers (VV0683, CM6.72b, CM6.75) and the indel marker (26749b) (S3 Table) were genotyped in fox F2 pedigrees which were previously genotyped with 18 SSRs assigned to VVU3. A high confidence map was constructed at a confidence level of 1000:1 (LOD ≥3) with crimap V2.504a [40]. Because GridQTL [41], the program used for QTL mapping, requires all markers to be placed at unique locations, the LOD 3.0 map was then saturated with unmapped SSRs regardless of the actual likelihood (LOD 0.0).

The indel marker (26749b) was also genotyped in 183 additional animals from three populations: tame (46), aggressive (45), and conventionally farm-bred foxes (92). The Fisher exact test (http://graphpad.com/quickcalcs/contingency2/) was used to evaluate the significance of the differences between each pair of populations.

### QTL analysis

Behavioral traits and phenotypes defined using PC analysis [12, 13, 25] were mapped in the F2 pedigrees using the F2inbred algorithm of GridQTL [41]. Permutation (n = 1,000) was used to establish chromosome-wide and experiment-wide significance thresholds.

## Results and Discussion

### Library sequencing

The *Eco*T22I GBS library sequenced using Illumina technology yielded a total of 35.5 Gbp of sequence data. After quality filtering (sequences without a barcode or restriction site remnant or with “Ns” were discarded) 20.8 Gbp of sequence was retained for analysis. On average, this dataset contained 5,202,895 reads of 100 bp length per sample (SD± 2,019,764; median: 4,517,500).

### SNP discovery and filtering

The GBS data were analyzed using the TASSEL-GBS pipeline to produce SNP calls [26]. A total of 2,003,563 tags (unique sequences) were identified and 1,530,295 (76.4%) of these aligned to single locations in the dog genome, 23,319 (1.2%) aligned to multiple positions, and 449,950 (22.5%) could not be aligned. The tags aligned to single locations in the dog genome produced a total of 101,940 SNP loci, of which 99,450 had exactly two alleles. Two sets of SNPs, filtered (48,294 SNPs, S4 Table) and stringently filtered (8,437 SNPs) sets were then produced. The amount of information obtained using GBS in foxes demonstrates the value of this approach for simultaneous SNP identification and genotyping in species without well-developed genomic tools.

### SNP evaluation

The SNP call reproducibility was evaluated using duplicated samples. In the stringently filtered set, the genotypes of replicates were in perfect concordance (the two replicates received the exact same calls) for 97.9–99.2% of SNPs among the seven duplicated samples.

In the filtered data set, the genotypes were in concordance 66.5–73.8% of the time. The disagreement was due to one sample not receiving a genotype call 55.6–71.7% of the time (i.e. missing data). The average depth for the discordant SNPs was 14.0 ± 25.4 reads, while for concordant SNPs the average depth was 34.0 ± 41.6 reads, thus the average depth for the concordant SNPs was 2.4 times greater than for the discordant ones (S5 Table). We hypothesized that many of the discordant SNPs are heterozygous SNPs where only one allele was captured. The analysis of the combined samples showed that 77.7–80.4% of discordant SNPs received heterozygous genotype calls in combined samples confirming that the greater depth increases the likelihood that both alleles are captured in heterozygous individuals. The analysis of the heterozygosity of SNPs located on X chromosome outside of PAR was consistent with these observations. The X chromosome SNPs from the filtered set showed increased heterozygosity in males (in average, 5.4% of males were heterozygous in each population) and decreased heterozygosity in females (in average, 12.4% of females in tame and 13.9% of females in aggressive population were heterozygous) (S5 Table). In contrast, the X chromosome SNPs from the stringently filtered set showed low level of heterozygosity in males (1.2% in tame and 1.0% in aggressive population) and relatively high heterozygosity in females (25.2% in tame and 29.4% in aggressive population) (S5 Table). We expect that increased heterozygosity in males largely reflects genotyping errors and low sequencing depth, while decreased heterozygosity in females reflects heterozygous under calling also due to not sufficient sequencing data. The high concordance and low level of heterozygosity of SNPs from X chromosome in males in the stringently filtered set are indicative of a low error rate among those SNPs.

### Assignment of SNP positions on fox chromosomes

The dog is the fox's closest relative with a sequenced genome. We used the comparative dog/fox map [13] and the known positions of fox SNPs in the dog genome to infer SNP positions on the fox chromosomes. Positions of 48,042 SNPs from the filtered SNP set (99.5% from the total number of SNPs in this set) and positions of 8,405 SNPs from the stringently filtered set (99.6% from the total number of SNPs in this set) were inferred in the fox.

### Comparative analysis of tame and aggressive fox populations using SNPs

#### Principal component analysis identified genetic divergence between the two populations

**Fig 1:**
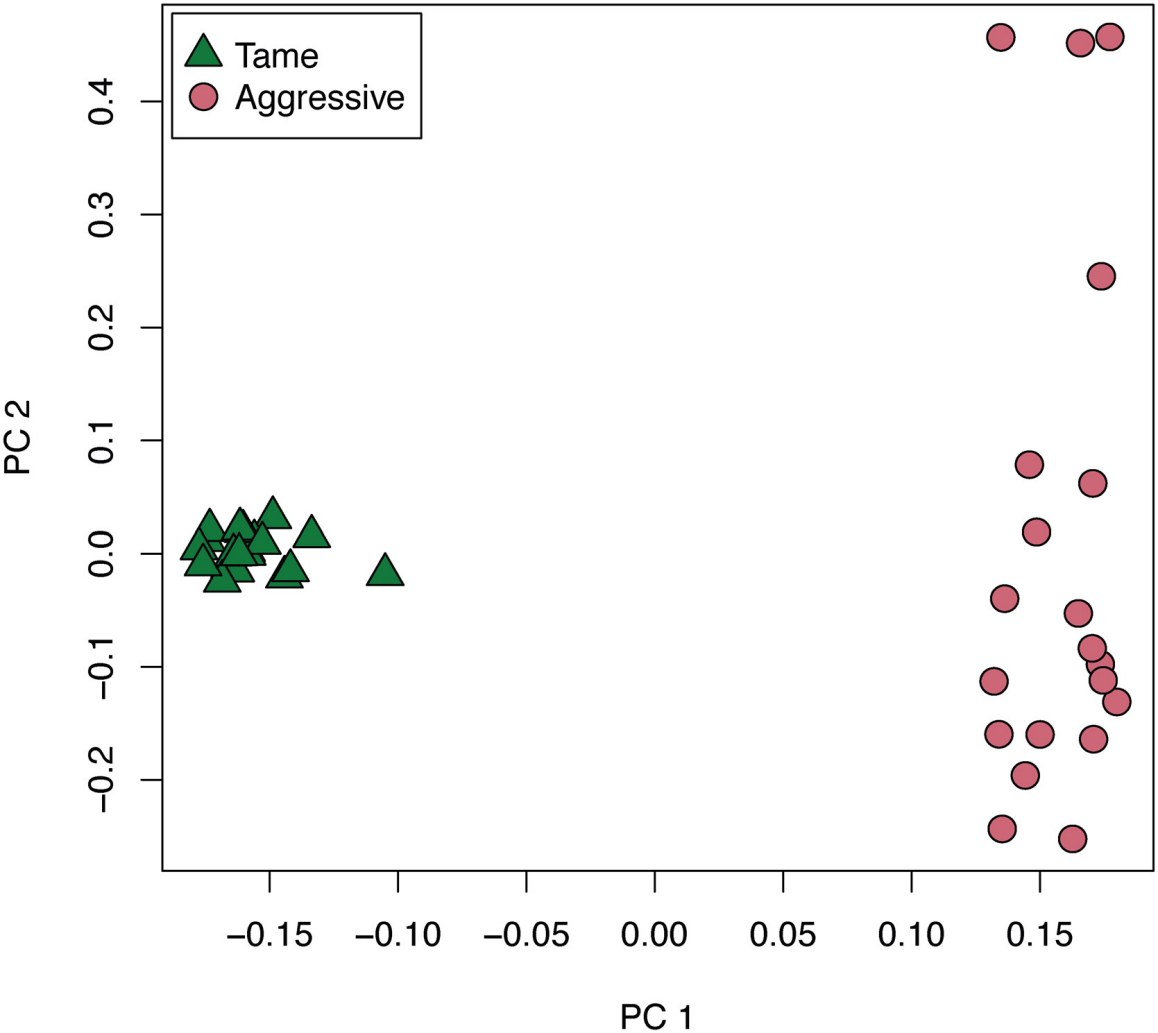
Principal component analysis. Principal component analysis of SNP data for 20 tame and 20 aggressive foxes. 8,437 SNPs with genotypes available for all individuals were used in this analysis. Aggressive individuals are represented by red dots, tame individuals are represented by green triangles. PC1 is plotted on the x-axis, PC2 is plotted on the y-axis.

The stringently filtered set of 8,437 SNPs with no missing data for any of the 40 individuals was subjected to Principal Component (PC) analysis. The PC analysis revealed genetic differences between the two strains (Fig 1). PC1 separated the samples into tame and aggressive groups, in agreement with the fact that the two populations have been maintained separately for more than 40 generations. PC2 identified more diversity in the aggressive strain than in the tame one, which may be attributable to the differences in history of the two populations. The tame population was maintained as a closed line through the breeding program, while the aggressive population experienced an introduction of foxes from conventionally farm-bred stock in the 1990s.

#### Estimation of the genetic structure using STRUCTURE

**Fig 2:**
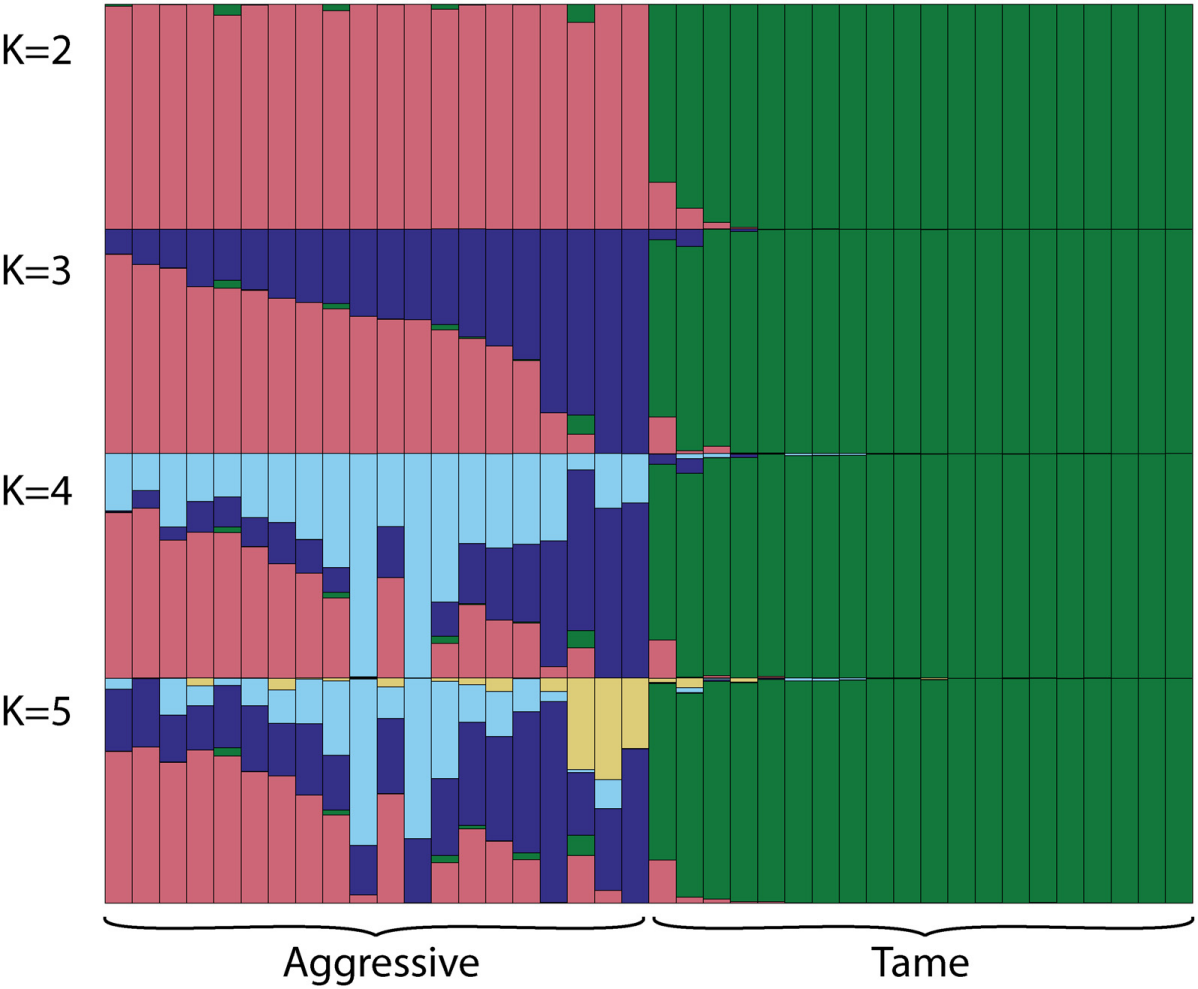
Estimation of population structure using STRUCTURE. Cluster analysis of fox genotypes was performed at four values of K (2, 3, 4, and 5) without population information. The numbers of assumed clusters are indicated on the y-axis. The population origin of individuals is indicated on x-axis. On each graph the individuals are listed in the order obtained at K = 3. Each individual is represented by a bar that is segmented into colors based on the assignment into inferred clusters given the assumption of K populations. The length of the colored segment is the estimated proportion of the individual’s genome belonging to that cluster. The analysis was run in 8 replicates for each K, the replicate with the highest likelihood is shown. The genetic structure analysis clearly differentiated the tame population from the aggressive one and did not reveal significant population stratification within the tame population at every K tested. In contrast, the population stratification within the aggressive population became apparent at K = 3.

The genetic structure analysis at K = 2 clearly separated the tame and aggressive individuals into two clusters (Fig 2). At K = 3 the stratification of the aggressive population became apparent. The further segmentation of the aggressive population was apparent at K = 4 and 5. Very little stratification was observed within the tame population at all K tested. The split of the aggressive population into subpopulation at K = 3 and higher Ks is consistent with the results of the PC analysis. In fact, the aggressive individuals which showed highest and lowest amount of segmentation based on the assignment into inferred clusters at K = 3, 4, and 5 had highest and lowest PC2 values, respectively. Both the PC analysis and the population structure analysis revealed the genetic diversification within the aggressive population which was likely caused by the admixture of the aggressive population with conventional population in 1990s.

#### Estimation of linkage disequilibrium and effective population size

**Fig 3:**
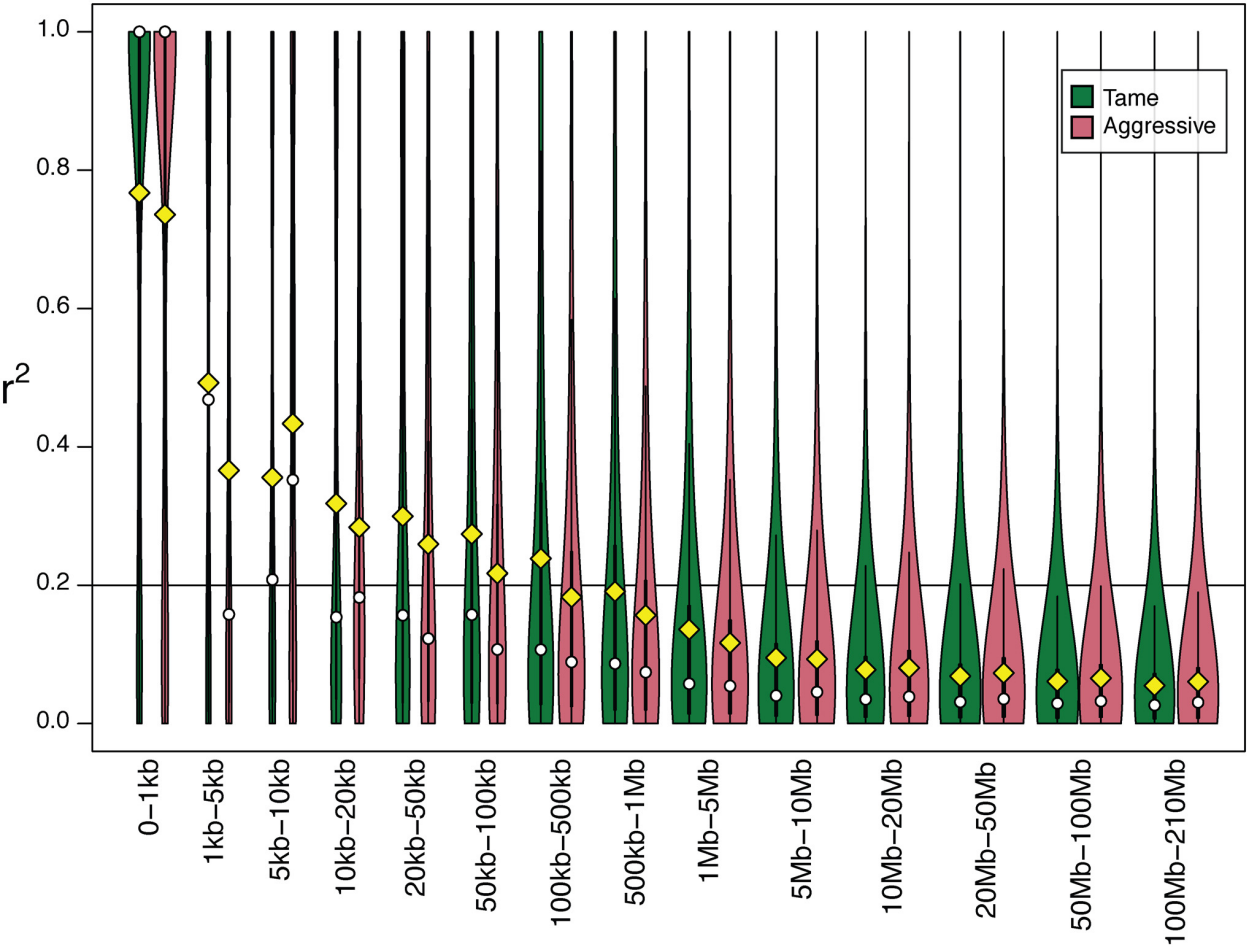
Estimation of linkage disequilibrium (r^2^) in tame and aggressive fox populations. Distributions of r^**2**^ values between pairs of SNPs separated by different distances are compared between tame (green) and aggressive (red) populations. SNP pairs were divided into 14 sets (bins) using the estimated distances between SNPs in the fox genome (S2 Table). Each bin is represented by a doubled bar (green and red) on the graph. The range of distances between SNPs in each bin is indicated on the x-axis. The width of the bar represents the relative number of SNP pairs in that bin for that population after a log transformation (wider bars have more pairs of SNPs). Exact numbers of SNP pairs in each bin are presented in S2 Table. The y-axis indicates r^**2**^ values for pairs of SNPs. The yellow diamonds correspond to the mean r^**2**^ for all SNPs in that bin in the population. The white circles correspond to the median values. The thin black line within each bar represents r^**2**^ values in that bin in the population in the interval from the 25th to 75th percentile. The horizontal line corresponds r^**2**^ = 0.2.

The relative order of and distances between SNPs in the fox genome were established by extrapolation from the dog genome. An average r^2^≥ 0.5 was observed for SNP pairs with inter-marker distance less than 1 Kb in both tame and aggressive populations. An average r^2^≥ 0.2 was observed for SNPs located within 100–500 Kb in tame and 50–100 Kb in the aggressive population (Fig 3, S2 Table). In general, the r^2^ values for SNPs in bins ranging from 1–1000 bp to 1Mb-5Mb were lower in the aggressive population than in the tame indicating a faster decay of LD in the aggressive population. The number of SNP pairs with r^2^ = 1 was slightly higher in tame than in the aggressive population (S2 Table). Less than 10% of SNP pairs with r^2^ = 1 was observed for SNPs located over 10 kb apart in the aggressive population and over 50 kb apart in the tame population (S2 Table).

To test that our data do not show significant spurious associations between unlinked SNPs the r^2^ was calculated for SNPs assigned to different fox chromosomes. In total, 22,184,914 pairs of SNPs in which each SNP in a pair was located on a different fox chromosome were analyzed in the tame and 27,725,667 pairs in the aggressive population. No substantial LD between unlinked SNPs was observed. Average r^2^ between SNPs located on different fox chromosomes was 0.055 (±0.079) in tame and 0.058 (±0.079) in aggressive populations.

Although estimation of the LD in fox strains could be influenced by the small sample size used in this study, the small number of SNPs, and imperfect calculation of intermarker distances based on fox/dog synteny, it still provides a useful indication of the extent of LD in these populations. The length of LD observed in the tame fox population is comparable with average LD length in dog breeds (r^2^ ≥ 0.2 was reported for SNPs located at the distance 500 Kb for several dog breeds [1]) and pigs (r^2^ = 0.15 and 0.2 were reported in two pig breeds for SNPs located 1 Mb apart [42]); and it is moderately higher than LD reported for two cow breeds (r^2^ = 0.3 was reported for SNPs located less than 100 kb in Holstein cattle and r^2^ = 0.25 for SNPs less than 20 kb in Fleckvieh breed [43–45]). The LD decay in cows is comparable with the LD profile in the aggressive fox strain. The length of LD in both fox strains is shorter than the length of LD in outbred mouse stock (r^2^ = 0.5 was reported for SNPs located 2 Mb apart [46]). The LD estimations calculated using sequence data has been reported to be lower than those calculated using SNP chip generated data, due to ascertainment bias of SNPs on the SNP chip [44]. The different technologies and MAF cut-offs between the studies makes the comparison between studies and species an imperfect estimation. The differences between our two populations, which were calculated from the same data, likely reflect the existing differences between the two strains.

Effective population sizes of the aggressive and tame populations, respectively, were 38.0 (95% CI: 37.8–38.2) and 81.5 (95% CI: 80.5–82.5). Although the aggressive population has a smaller number of breeding animals and lower effective population size, more genetic variation has been preserved in this population. These findings may reflect the fact the aggressive population had an inclusion of outside individuals in the recent past in comparison to the tame population, which experienced a longer period of closed breeding.

### Comparison of SNP allele frequencies between tame and aggressive fox populations

#### Analysis of the allele frequency differences between the two populations

**Fig 4:**
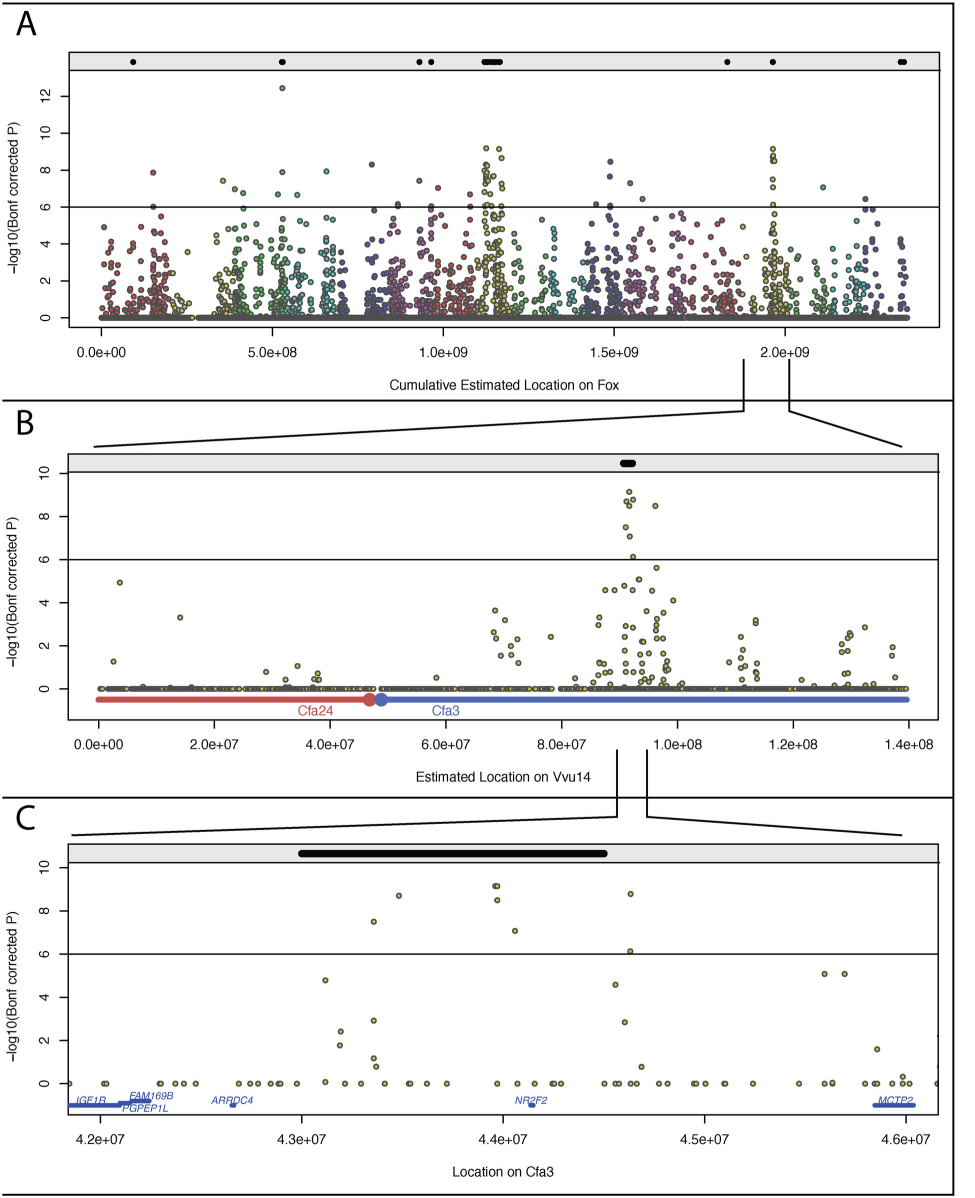
Allele frequency differences of genome-wide and VVU14 SNPs. The black dots within the gray bar on the top of each figure panel indicate regions with F_ST_≥0.5. Dots of different colors are the Bonferroni corrected significance of the allele frequency differences between the tame and aggressive populations calculated by PLINK2. The y-axis indicates-log10 (Bonferroni corrected p-value) for SNP allele frequency differences. The black horizontal line corresponds to a significance cutoff of (-log10 = 6). Genomic position in fox, as extrapolated from syntenic regions in the dog genome, is plotted on the x-axis. **4A.** Differences in allele frequency of SNPs genome wide. Colors indicate the different fox chromosomes. The x-axis indicates the cumulative estimated positions in the fox genome in megabases. **4B.** Differences in allele frequency of SNPs on VVU14. The horizontal colored bars on the x-axis correspond to syntenic dog chromosomes. **4C.** Multi SNPs region on VVU14 with significant allele frequency differences between the two populations. CFA3:42,000,000–46,000,000 corresponding to the cluster on VVU14 with multiple SNPs that are highly significant. The genes in the region are indicated just above the x-axis. The gene *NR2F2* (*COUP-TFII*) is the only gene located in the significant region.

Overall, a relatively high level of heterozygosity was observed in both populations (Table 1), clearly demonstrating that efforts to limit inbreeding in these strains has been effective. A slightly lower level of heterozygosity was observed in the tame population than in the aggressive one (mean expected heterozygosity in tame population was 0.22 versus 0.24 in the aggressive population). The average SNP minor allele frequency was slightly lower in the tame strain than in the aggressive strain and a higher percent of SNPs was monomorphic in the tame than in the aggressive strain (Table 1). Comparison of allele frequencies between the two populations (Fig 4a, Table 2) identified 68 SNPs on 12 fox chromosomes that showed significant differences in allele frequency (Bonferroni correction of association test; p<10^-7^). These included 19 “isolated” SNPs (> 2Mb from another significant SNP), and 49 SNPs located within 11 clusters on seven fox chromosomes. Clusters comprised at least two SNPs located within a 2 Mb interval (Table 2). To further characterize these genomic intervals, genes located inside or within 50,000 bp from start and end of these regions were identified based on dog genome annotations (Table 3). Several of these intervals were found to include genes involved in neurological functioning and behavior (Table 3).

**Table 1:** SNP minor allele frequency and heterozygosity in tame and aggressive populations.

Population	Number of SNPs	Mean MAF	Median MAF	SD MAF	Fixed in the population	Mean Expected Heterozygosity	Mean Observed SNP Heterozygosity	Median Observed SNP Heterozygosity	SD SNP Heterozygosity
Tame	48,294	0.148	0.088	0.147	8,014	0.22	0.16	0.10	0.16
Aggressive	48,294	0.163	0.115	0.147	5,514	0.24	0.17	0.11	0.16

**Table 2:** SNPs with significant allele frequency differences between tame and aggressive populations.

SNP	CFA	Location on CFA	VVU	Inferred location on VVU	Differences of SNP minor allele frequencies between two populations	Bonferroni corrected P-value	Distance from previous significant SNP	Clusters
**S1_285236887**	**12**	**18,837,258**	**1**	**167,384,431**	**0.80**	**1.36E-08**		**1**
**S1_285704585**	**12**	**19,304,956**	**1**	**167,852,129**	**0.82**	**9.59E-07**	**467,698**	**1**
S1_812259209	2	51,452,767	2	138,634,343	0.80	3.76E-08		
S1_1648737836	36	1,743,329	3	1,743,329	0.79	1.08E-07		
S1_1673084403	36	26,089,896	3	26,089,896	0.77	1.74E-07	24,346,567	
S1_1964707411	6	54,892,000	3	127,827,426	0.75	2.05E-07	101,737,530	
**S1_1977727319**	**6**	**67,911,908**	**3**	**140,847,334**	**0.82**	**1.28E-08**	**13,019,908**	**2**
**S1_1977727423**	**6**	**67,912,012**	**3**	**140,847,438**	**0.97**	**3.58E-13**	**104**	**2**
**S1_1977727436**	**6**	**67,912,025**	**3**	**140,847,451**	**0.82**	**1.28E-08**	**13**	**2**
S1_1531784156	32	23,626,827	4	42,200,559	0.88	2.20E-07		
S1_1794484892	4	61,861,562	4	127,688,948	0.86	1.16E-08	85,488,389	
S1_1712828536	38	4,119,843	5	105,433,843	0.82	4.94E-09		
**S1_986356551**	**22**	**31,130,422**	**6**	**31,130,422**	**0.77**	**6.91E-07**		**3**
**S1_987024936**	**22**	**31,798,807**	**6**	**31,798,807**	**0.75**	**8.95E-07**	**668,385**	**3**
**S2_113588644**	**8**	**32,614,012**	**6**	**94,053,946**	**0.80**	**3.76E-08**	**62,255,139**	**4**
**S2_113588820**	**8**	**32,614,188**	**6**	**94,054,122**	**0.80**	**3.76E-08**	**176**	**4**
S2_148636413	8	67,661,781	6	129,101,715	0.75	8.95E-07	35,047,593	
S1_540429371	16	13,131,693	7	13,131,693	0.7750	9.22E-08		
**S1_449099782**	**14**	**46,959,949**	**7**	**106,592,795**	**1.0000**	**2.05E-07**	**93,461,102**	**5**
**S1_449883141**	**14**	**47,743,308**	**7**	**107,376,154**	**0.7500**	**9.48E-07**	**783,359**	**5**
**S1_1235603545**	**27**	**28,350,100**	**8**	**28,350,100**	**0.8417**	**1.05E-08**		***6***
**S1_1235712667**	**27**	**28,459,222**	**8**	**28,459,222**	**0.8973**	**1.52E-07**	**109,122**	**6**
**S1_1236925680**	**27**	**29,672,235**	**8**	**29,672,235**	**0.7500**	**8.95E-07**	**1,213,013**	**6**
**S1_1238406667**	**27**	**31,153,222**	**8**	**31,153,222**	**0.7750**	**5.42E-08**	**1,480,987**	**6**
**S1_1240026884**	**27**	**32,773,439**	**8**	**32,773,439**	**0.8000**	**3.09E-08**	**1,620,217**	**6**
**S1_1240026888**	**27**	**32,773,443**	**8**	**32,773,443**	**0.8250**	**4.94E-09**	**4**	**6**
**S1_1240026889**	**27**	**32,773,444**	**8**	**32,773,444**	**0.8250**	**4.94E-09**	**1**	**6**
**S1_1240026890**	**27**	**32,773,445**	**8**	**32,773,445**	**0.8250**	**4.94E-09**	**1**	**6**
**S1_1240254310**	**27**	**33,000,865**	**8**	**33,000,865**	**0.8000**	**2.20E-08**	**227,420**	**6**
**S1_1240254354**	**27**	**33,000,909**	**8**	**33,000,909**	**0.8000**	**2.20E-08**	**44**	**6**
**S1_1240725455**	**27**	**33,472,010**	**8**	**33,472,010**	**0.8184**	**1.81E-08**	**471,101**	**6**
**S1_1240725498**	**27**	**33,472,053**	**8**	**33,472,053**	**0.8184**	**1.81E-08**	**43**	**6**
**S1_1240736757**	**27**	**33,483,312**	**8**	**33,483,312**	**0.8711**	**6.52E-10**	**11,259**	**6**
**S1_1242777422**	**27**	**35,523,977**	**8**	**35,523,977**	**0.8591**	**1.34E-08**	**2,040,665**	**7**
**S1_1242825122**	**27**	**35,571,677**	**8**	**35,571,677**	**0.8000**	**3.09E-08**	**47,700**	**7**
**S1_1243526396**	**27**	**36,272,951**	**8**	**36,272,951**	**0.9118**	**5.75E-09**	**701,274**	**7**
**S1_1243526433**	**27**	**36,272,988**	**8**	**36,272,988**	**0.9118**	**5.75E-09**	**37**	**7**
**S1_1243639565**	**27**	**36,386,120**	**8**	**36,386,120**	**0.8000**	**4.02E-08**	**113,132**	**7**
**S1_1243639695**	**27**	**36,386,250**	**8**	**36,386,250**	**0.7500**	**7.52E-07**	**130**	**7**
S1_1246055552	27	38,802,107	8	38,802,107	**0.8000**	3.76E-08	2,415,857	
S1_1252424198	27	45,170,753	8	45,170,753	**0.7750**	1.97E-07	6,368,646	
S1_648198405	17	61,267,781	8	48,897,988	**0.8733**	8.80E-07	3,727,235	
S1_644111987	17	57,181,363	8	52,984,406	**0.8000**	2.20E-08	4,086,418	
S1_641943154	17	55,012,530	8	55,153,239	**0.8250**	2.08E-08	2,168,833	
S1_625871222	17	38,940,598	8	71,225,171	**0.8500**	7.10E-10	16,071,932	
**S1_619211128**	**17**	**32,280,504**	**8**	**77,885,265**	**0.7500**	**8.95E-07**	**6,660,094**	**8**
**S1_618721739**	**17**	**31,791,115**	**8**	**78,374,654**	**0.9063**	**2.22E-09**	**489,389**	**8**
**S1_618399870**	**17**	**31,469,246**	**8**	**78,696,523**	**0.7750**	**5.42E-08**	**321,869**	**8**
**S1_616717168**	**17**	**29,786,544**	**8**	**80,379,225**	**0.8404**	**9.78E-08**	**1,682,702**	**8**
**S1_922966764**	**21**	**18,599,358**	**11**	**32,259,265**	**0.8125**	**7.05E-07**		**9**
**S1_922966746**	**21**	**18,599,340**	**11**	**32,259,283**	**0.8125**	**7.05E-07**	**18**	**9**
**S1_1038102049**	**23**	**21,435,886**	**11**	**72,294,509**	**0.8000**	**2.20E-08**	**40,035,226**	**10**
**S1_1039257018**	**23**	**22,590,855**	**11**	**73,449,478**	**0.8000**	**8.32E-07**	**1,154,969**	**10**
**S1_1039763208**	**23**	**23,097,045**	**11**	**73,955,668**	**1.0000**	**3.49E-09**	**506,190**	**10**
S1_237429054	11	45,418,622	12	28,970,475	0.8111	5.07E-08		
S1_201401022	11	9,390,590	12	64,998,507	0.7500	3.64E-07	36,028,032	
**S1_1379515115**	**3**	**43,357,310**	**14**	**91,056,089**	**0.8750**	**3.16E-08**		**11**
**S1_1379640157**	**3**	**43,482,352**	**14**	**91,181,131**	**0.8421**	**1.98E-09**	**125,042**	**11**
**S1_1380118429**	**3**	**43,960,624**	**14**	**91,659,403**	**0.8500**	**7.10E-10**	**478,272**	**11**
**S1_1380118781**	**3**	**43,960,976**	**14**	**91,659,755**	**0.8500**	**7.10E-10**	**352**	**11**
**S1_1380128532**	**3**	**43,970,727**	**14**	**91,669,506**	**0.8250**	**3.20E-09**	**9,751**	**11**
**S1_1380128533**	**3**	**43,970,728**	**14**	**91,669,507**	**0.8500**	**7.10E-10**	**1**	**11**
**S1_1380128577**	**3**	**43,970,772**	**14**	**91,669,551**	**0.8500**	**7.10E-10**	**44**	**11**
**S1_1380216459**	**3**	**44,058,654**	**14**	**91,757,433**	**0.8000**	**8.43E-08**	**87,882**	**11**
**S1_1380788560**	**3**	**44,630,755**	**14**	**92,329,534**	**0.8889**	**7.45E-07**	**572,101**	**11**
**S1_1380790456**	**3**	**44,632,651**	**14**	**92,331,430**	**0.8824**	**1.64E-09**	**1,896**	**11**
S1_1384636870	3	48,479,065	14	96,177,844	0.8250	3.20E-09	3,846,414	
S1_1272088483	28	18,958,228	15	99,068,409	0.7921	8.52E-08	2,890,565	

SNPs with differences in allele frequency between two populations at p <10^-7^ (Bonferroni correction of PLINK2 [32] association test). The absolute differences of SNP minor allele frequencies between the tame and aggressive populations are listed. These SNPs are presented as dots above the significance line (–log10 >6) on Fig 4. SNPs are listed in the order of the fox map. Multi SNP clusters are in bold and numbered in the rightmost column. Multi SNP clusters are regions where SNPs that met the threshold are within 2 Mb to the next significant SNP. Genes that are in or near (50,000 bp) the multi SNP clusters are listed in Table 3. Dog chromosome—CFA; Fox chromosome—VVU.

**Table 3:** Genes located inside or within 50,000 bp from start and end of the multi SNP clusters in the dog genome.

Cluster	Genes
1	*PKHD1*
2	none
3	*SCEL*, *SLAIN1*, *EDNRB*
4	*RPL17*, *(OTX2-AS1*
5	*TBX20*, *HERPUD2*, *SEPT7*, *EEPD1*, *KIAA0895*
6	*CAPZA3*, *PLCZ1*, *PIK3C2G*, *RERGL*, *LMO3*, *MGST1*, *SLC15A5*, *DERA*, *STRAP*, *EPS8*, *PTPRO*, *RERG*, *PDE6H*, *ARHGDIB*, *ERP27*, *MGP*, *ART4*, *C27H12orf60*, *SMCO3*, *WBP11*, *H2AFJ*, *TRNAG-UCC*, *GUCY2C*, *PLBD1*, *ATF7IP*, *GRIN2B*, *EMP1*, *GSG1*, *KIAA1467*, *HEBP1*, *GPRC5D*, *GPRC5A*, *DDX47*
7	*KLRC1*, *KLRK1*, *KLRD1*, *GABARAPL1*, *TMEM52B*, *OLR1*, *CLEC7A*, *CLEC1B*, *CLEC12B*, *CLEC12A*, *KLRF2*, *CLEC2B*, *KLRF1*, *CD69*, *CLEC2D*, *KLRB1*, *KLRB1*
8	*QPCT*, *CDC42EP3*, *FAM82A1*, *CYP1B1*, *ATL2*, *HNRNPLL*, *GALM*, *SRSF7*, *GEMIN6*, *DHX57*, *MORN2*, *ARHGEF33*, *SOS1*, *CDKL4*, *MAP4K3*, *TMEM178A*, *THUMPD2*, *SLC8A1*
9	*OFD1*
10	*ZNF385D*, *TEAD2*, *SGOL1*
11	*COUP-TFII* (*NR2F2*)

Cluster numbers refer to the numbering from Table 2.

#### The fixation index analysis

The weighted fixation index analysis (F_ST_) of fox populations identified 28 windows with F_ST_ ≥ 0.5 (S2 Fig, S6 Table, S3 Fig). The windows with high F_ST_ values clustered into nine regions in the fox genome (Fig 4a; S6 Table). Out of 18 windows with F_ST_ ≥ 0.5 identified on fox chromosome 8 (VVU8) 14 windows overlap with the genomic regions identified in the analysis of allele frequency differences (S3 Fig). Four windows with high F_ST_ values (S6 Table) which were identified on VVU6 and VVU14 also overlap with the regions identified in the analysis of allele frequency differences (Fig 4a).

### Analysis of genomic regions on VVU3, VVU8 and VVU14 in fox crossbred pedigrees

Genomic regions with increased divergence between the tame and aggressive populations could represent regions influenced by either behavioral selection or random fixation (genetic drift). To distinguish between these processes, we hypothesized that regions representing true selection targets would be identified by both selective sweep mapping in fox populations and QTL mapping of behavioral traits in fox experimental pedigrees. Regions influenced by random fixation, on the other hand, would be identified only by homozygosity mapping, but would not co-segregate with behavioral phenotypes in informative pedigrees. To test this hypothesis, three genomic regions on fox chromosomes 3 (VVU3), 8 (VVU8) and 14 (VVU14), which were among the regions which showed the greatest allele frequency differences between the two populations (Table 2; Fig 4; S2 Fig, S6 Table; S3 Fig), were examined in more detail.

#### Distal region of VVU3

The distal part of VVU3 contains a genomic region that appears to be approaching fixation for different alleles in the tame and aggressive populations. Three SNPs located within a 117 bp interval showed significant differences in allele frequencies including SNP-S1_1977727423, which showed the most significant differences in the Bonferroni corrected association test among all fox SNPs. The SNP-S1_1977727423 was monomorphic in the aggressive population while the frequency of the only allele observed in the aggressive population was 0.03 in the tame population. The allele frequencies for two other SNPs (SNP-S1_1977727319 and SNP-S1_1977727436) were the same for both SNPs: the minor allele frequency for these SNPs was 0.85 in the aggressive and 0.03 in the tame population. The distal region of VVU3 had been excluded from previous QTL mapping studies because this region has not been represented on the fox meiotic linkage map.

Allele frequency differences on distal VVU3 were validated in an additional sample set comprising tame (46), aggressive (45), and conventionally farm-bred foxes (92) using an indel marker, 26749b, located about 122 Kb from SNP-S1_1977727423. Details of this analysis are presented in S1 File. The Fisher exact test analysis identified significant differences in indel allele frequencies between tame and aggressive, and between tame and conventionally bred foxes; in both cases a significance level of p <0.0001 was observed. No significant difference was observed between aggressive and conventional farm-bred foxes (p = 0.5693). Assuming that the conventionally bred population is similar to the ancestral population for both tame and aggressive populations, these results suggest that the relevant genomic region on VVU3 has been under intense selection or drift in the tame population but not in the aggressive population.

To evaluate the effect of distal region of VVU3 on fox behavior we extended the meiotic linkage map for VVU3 [13] using new dog-derived SSR and indel markers (S3 Table) by 38.4 cM (S4 Fig). However, the QTL analysis using this new VVU3 map did not identify any significant behavioral QTL in F2 pedigrees in the region of interest. These results suggest that the distal region on VVU3 may represent an example of random allele fixation in a closed population (i.e. genetic drift) rather than a selective sweep associated with selection for behavior. Alternatively, we cannot rule out the possibility that the behavioral assay in these populations does not capture some behavioral or even physiological parameters important for expression of tame or aggressive behavior. A more thorough analysis is needed to exclude this candidate region as a region involved in regulation of behavior.

#### VVU8

VVU8 contains 29 out of the 68 SNPs which showed significant differences in allele frequency between the tame and aggressive strains. The region of genomic divergence on VVU8 includes three of the 11 SNP clusters. It is estimated to be about 52 Mb in the fox genome and spans two different dog chromosomes, CFA27 and CFA17. In this extended region there are also many SNPs that do not reach our threshold, but show a high level of allele frequency differences between the tame and aggressive populations (Fig 4a; S3 Fig). Comparison of this region with previously identified QTLs for PC defined behavioral phenotypes did not identify any significant overlap. However, suggestive QTLs for several individual behavioral traits were identified in this area of VVU8 including the QTL for the trait “Attack” [25] (Nelson et al., in preparation), therefore this region requires further evaluation. While it is difficult to draw firm conclusions or find specific genes in such a large area, some genes, notably *GRIN2B* (*NMDA*) with GO terms including (http://amigo.geneontology.org/) “startle response” and “behavioral fear response” and *GABARAPL1* (*GEC1*), which enhances expression of the kappa opioid receptor [47], both stand out as possible candidate genes.

#### VVU14

Comparison of allele frequencies between tame and aggressive individuals identified 11 SNPs on fox chromosome 14 (VVU14) which met our significance threshold, 10 of which are grouped tightly within a 1.3 Mb region (Fig 4, Table 2). Previously, we identified several significant QTLs for PC defined behavioral phenotypes and individual behavioral traits on VVU14 [25] (Nelson et al., in preparation). The behavioral QTL intervals include the 1.3 Mb region identified in this study. This region on VVU14 corresponds to the dog region CFA3:43,357,310–44,632,651 bp and contains one gene, *COUP-TFII (NR2F2)*. COUP-TFII is an orphan nuclear receptor belonging to the superfamily of steroid/thyroid hormone receptors [48]. COUP-TFII expression during embryonic development is significant for the development of forebrain and several other brain regions including amygdala [48–50]. It has been shown that COUP-TFII can also act as a silencer of the human oxytocin gene promoter *in vitro* [51]. *COUP-TFII* is an interesting candidate gene and its role in regulation of behavior in foxes will be further evaluated.

Identification of loci under selection in artificially selected populations and natural bottlenecked populations remains to be a challenge [52, 53]. Results of the selective sweep analysis in such populations can be influenced by population demographic histories and reduced effective population sizes [54–56]. Application of a combination of methods including selective sweep mapping in populations under selection and QTL analysis of informative pedigrees represents a promising strategy for differentiation between the signals of selection and random fixation and disentangling genetic architecture of phenotypes under selection [57–62].

The pilot analysis of the genetic structure of tame and aggressive fox strains using GBS clearly shows the potential for using these strains for high-resolution genetic mapping of behavioral phenotypes. The genetic structure and the effective population size of tame and aggressive strains indicate that a significant amount of genetic diversity has been preserved in both populations. Simultaneously, intensive selection for behavior in these populations must favor the accumulation of specific alleles in the targeted regions of selection. Understanding genetic composition of these populations will facilitate the use of this unique animal model for studying the genetics of social interactive behavior. The current analysis confirmed loci previously determined to be under selection [12] (Nelson et al., in preparation) and pinpointed novel genomic regions to be investigated for their role in regulation of behavior.

## Conclusions

The first analysis of fox populations using genome-wide distributed SNPs (48,294 SNPs) revealed the genetic structure of the tame and aggressive strains and identified several genomic regions with significantly different allele frequencies between the two populations. The population genomic parameters of the fox strains clearly indicated the deep potential of these strains for high resolution mapping of behavior. The growth of sequencing technologies will allow us to search for signals of selection in these fox strains by whole genome sequencing. The VVU14 region with high allele frequency differences between the two strains overlaps with a previously identified QTL for behavior in fox experimental pedigrees emphasizing the importance of this region for behavior. Comparative analysis of selective sweep positions and QTL intervals can be used as a promising approach for differentiation between selective sweeps associated with selection and regions of random fixation [19, 60]. Identification of selective sweeps associated with QTLs will be highly advantageous for selection of positional candidate genes. The GBS protocol developed in this study can be easily adapted for genomic studies of other canids.

## Supporting Information

S1 Fig Details about enzyme selection and exclusion. (PDF)

S2 Fig Fixation index (F_ST_) analysis in fox populations. (PDF)

S3 Fig Allele frequency differences and significant F_ST_ windows on VVU8. (PDF)

S4 Fig Meiotic linkage map of VVU3. (PDF)

S1 File Genotyping of 26749b marker in three fox populations. (PDF)

S1 Table Adapters used for construction of *Eco*T22I library. (PDF)

S2 Table Bins used for calculation of average r^2^ between SNPs located on the same chromosome. (PDF)

S3 Table Florescent primers used for genotyping S1_1977727423 associated indels and SSRs markers on VVU3. (PDF)

S4 Table The list of fox SNPs and their locations in the dog genome. (XLSX)

S5 Table SNP evaluation statistics. (PDF)

S6 Table Genomic regions highlighted in the fixation index (F_ST_) analysis. (PDF)
