# Supplementary material for: Genotyping-By-Sequencing (GBS) Detects Genetic Structure and Confirms Behavioral QTL in Tame and Aggressive Foxes (Vulpes vulpes)
Source: PLoS One. 2015 Jun 10;10(6):e0127013. doi: 10.1371/journal.pone.0127013 (PMC4465646; doi:10.1371/journal.pone.0127013)
Supplement: S1 Fig — (PDF) [file pone.0127013.s001.pdf]

Figure S1. Fragment size distribution of the silver fox GBS libraries prepared using different restriction enzymes. Experion® output showing profiles for single DNA sample libraries prepared using one restriction enzyme: *Eco*T22I (**A**), *Ape*KI (**B**), *Pst*I (**C**) or two enzymes: *Eco*T22I and *Pst*I (**D**). The x-axis denotes elution time in seconds and the y-axis shows fluorescence units. Two discrete peaks (15 and 1500 bp) correspond to the size standard. The size of fragments corresponding to the broad peaks (library) are indicated above hatch marks. Sharp peaks at 227 bp observed in the libraries prepared with *Pst*I (C) or in double digest with *Eco*T22I and *Pst*I (D) most likely correspond to repetitive sequence. Small peak around 45 seconds (~70bp) corresponds to PCR primer dimers.

A **B**

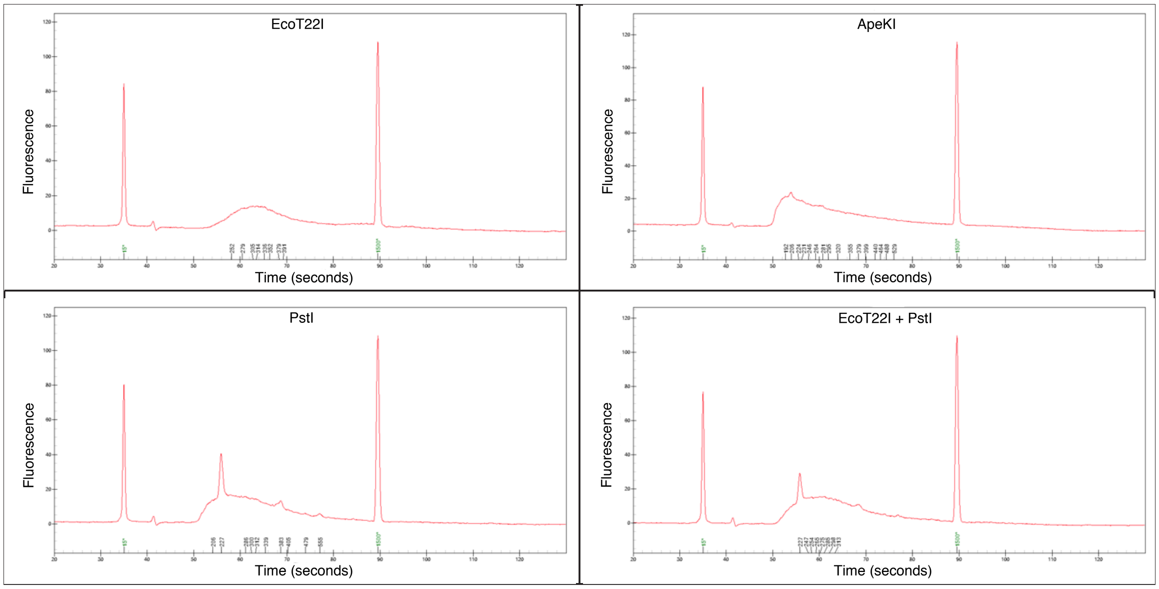

C **D**
