# Supplementary material for: Genotyping-By-Sequencing (GBS) Detects Genetic Structure and Confirms Behavioral QTL in Tame and Aggressive Foxes (Vulpes vulpes)
Source: PLoS One. 2015 Jun 10;10(6):e0127013. doi: 10.1371/journal.pone.0127013 (PMC4465646; doi:10.1371/journal.pone.0127013)
Supplement: S2 Fig — (PDF) [file pone.0127013.s002.pdf]

Figure S2. Fixation index (F_{ST}) analysis in fox populations.

Fixation index (F_{ST}) values were computed for 1 Mb windows for tame versus aggressive fox populations. The y-axis indicates "weighted_F_{ST}" values calculated with VCFtools. The x-axis indicates genomic positions on fox chromosomes, as extrapolated from syntenic regions in the dog genome. Each dot represents a 1 Mb window, the windows overlap by 500 kb. The horizontal black line indicates F_{ST}=0.5. The results of the F_{ST} analysis are consistent with the analysis of allele frequency differences.

S2A. **Distribution of F_{ST} values in the fox genome.** The chromosomes are indicated by color change. The positions of VVU3, VVU8 and VVU14 are shown.

S2B. F_{ST} values on VVU3, VVU8, and VVU14. The regions with high F_{ST} values identified on these chromosomes overlap with the regions identified in the analysis of allele frequency differences (see also Table S6).

A.

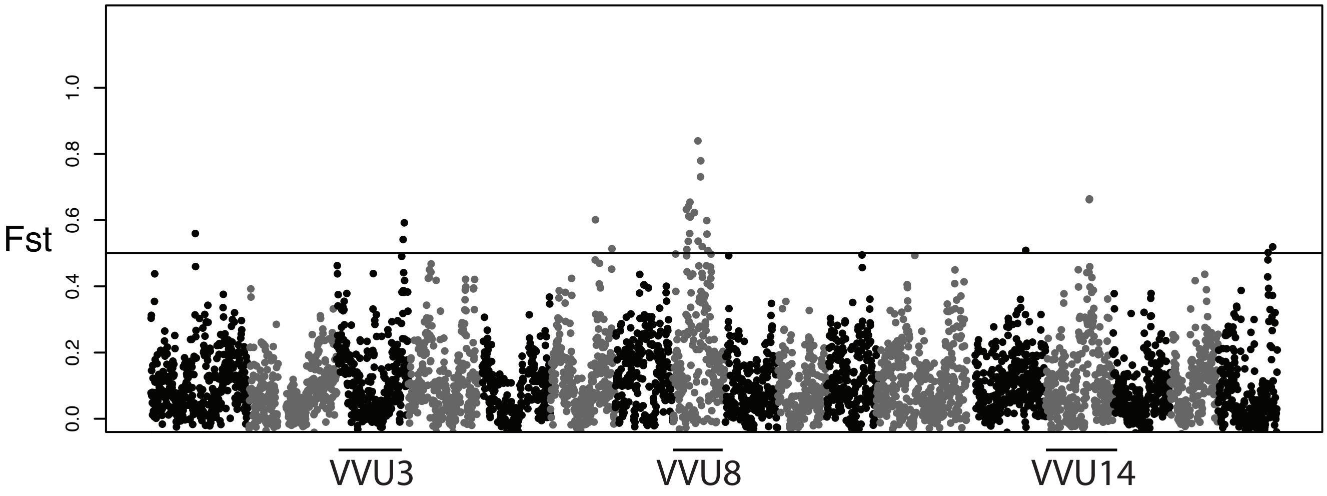

B.

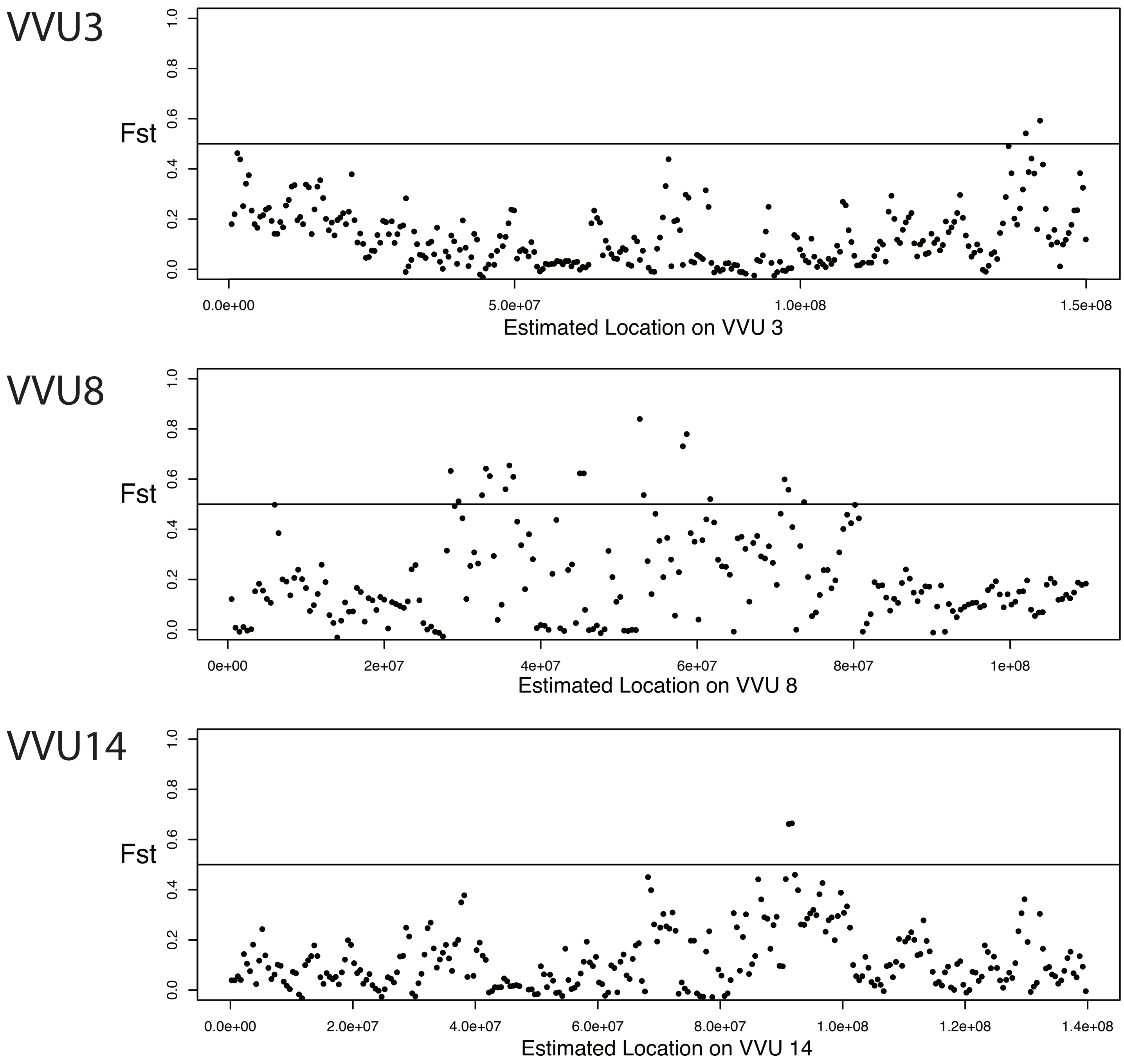

VVU3
Fst
1.0
0.8
0.6
0.4
0.2
0.0
0.0e+00
5.0e+07
1.0e+08
1.5e+08
Estimated Location on VVU 3
VVU8
Fst
1.0
0.8
0.6
0.4
0.2
0.0
0e+00
2e+07
4e+07
6e+07
8e+07
1e+08
Estimated Location on VVU 8
VVU14
Fst
1.0
0.8
0.6
0.4
0.2
0.0
0.0e+00
2.0e+07
4.0e+07
6.0e+07
8.0e+07
1.0e+08
1.2e+08
1.4e+08
Estimated Location on VVU 14
