# Supplementary material for: Genotyping-By-Sequencing (GBS) Detects Genetic Structure and Confirms Behavioral QTL in Tame and Aggressive Foxes (Vulpes vulpes)
Source: PLoS One. 2015 Jun 10;10(6):e0127013. doi: 10.1371/journal.pone.0127013 (PMC4465646; doi:10.1371/journal.pone.0127013)

Figure S3. **Allele frequency differences and significant FST windows on VVU8.**

The black dots within the gray bar on the top indicate regions with FST≥0.5. Yellow dots are the Bonferroni corrected significance of the allele frequency differences between the tame and aggressive populations calculated by PLINK2. The y-axis indicates -log10 (Bonferroni corrected p-value) for SNP allele frequency differences. Genomic positions in fox, as extrapolated from syntenic regions in the dog genome, are plotted on the x-axis. The black horizontal line corresponds to a significance cutoff of (-log10 = 6) for the allele frequency differences. The horizontal colored bars on the x-axis correspond to syntenic dog chromosomes. Two genes, *GRIN2B (NMDA)* and *GABARAPL1 (GEC1),* that represent interesting positional candidate genes are shown.

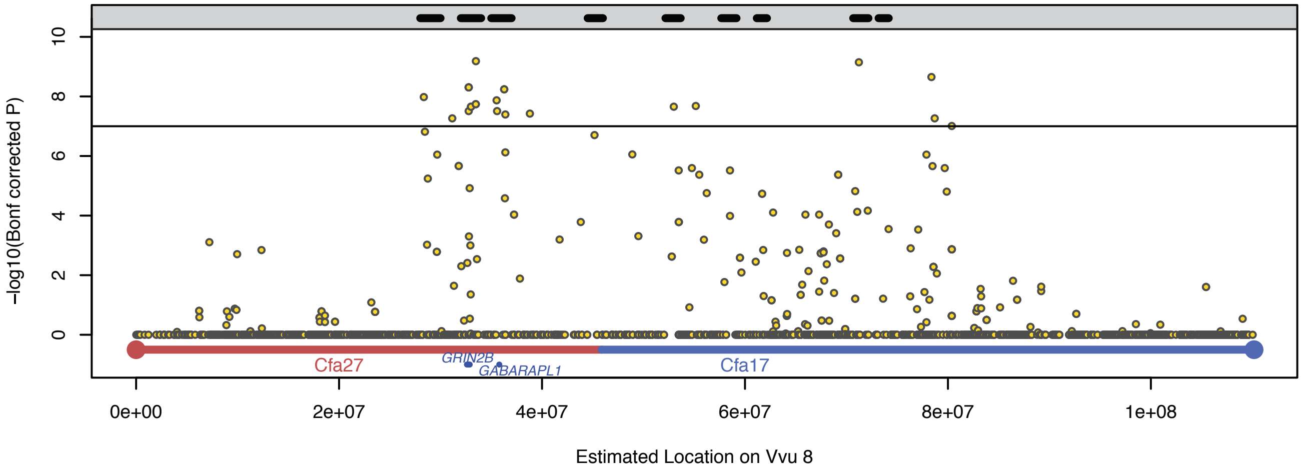

Supplement: S3 Fig — (PDF) [file pone.0127013.s003.pdf]
