# Supplementary material for: Genotyping-By-Sequencing (GBS) Detects Genetic Structure and Confirms Behavioral QTL in Tame and Aggressive Foxes (Vulpes vulpes)
Source: PLoS One. 2015 Jun 10;10(6):e0127013. doi: 10.1371/journal.pone.0127013 (PMC4465646; doi:10.1371/journal.pone.0127013)

Figure S4. Meiotic linkage map of VVU3

The markers were placed on the map at a confidence of LOD 3. Markers that were not placed in on the map at this confidence level are indicated in their most likely positions along the right side of the map.

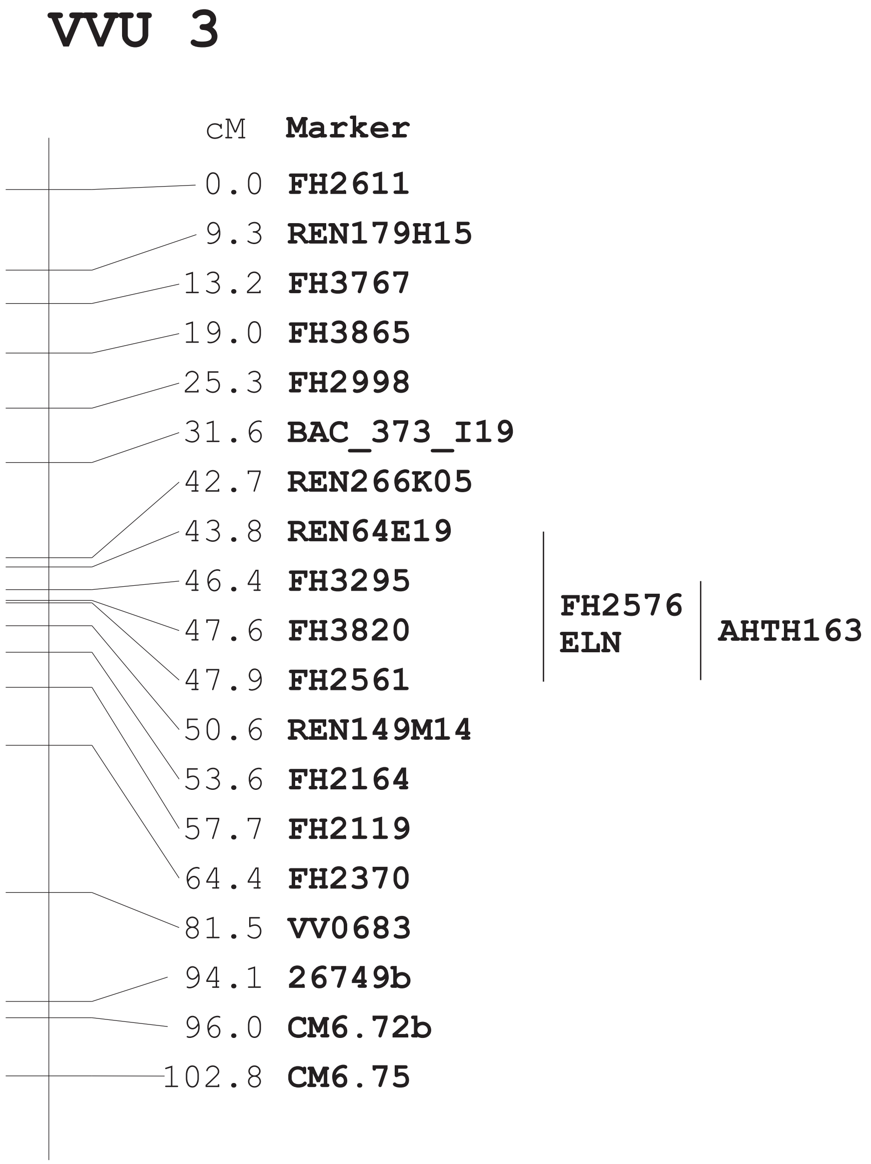

Supplement: S4 Fig — (PDF) [file pone.0127013.s004.pdf]
