# Supplementary material for: Genotyping-By-Sequencing (GBS) Detects Genetic Structure and Confirms Behavioral QTL in Tame and Aggressive Foxes (Vulpes vulpes)
Source: PLoS One. 2015 Jun 10;10(6):e0127013. doi: 10.1371/journal.pone.0127013 (PMC4465646; doi:10.1371/journal.pone.0127013)
Supplement: S1 File — (PDF) [file pone.0127013.s005.pdf]

File S1. Genotyping of 26749b marker in three fox populations

An amplicon located approximately 122 Kb from SNP S1_1977727423 was amplified with primers 26749F-CCTCTGCTCATCCACAGTCG and 26749R-CGCCCTAAGGAGCTGAGTGT which were designed with Primer3 using CanFam3 as a template. Analysis of the amplicon in 4 tame (Tame), 6 aggressive (Aggr), and 9 conventional farm-bred foxes (Conv) identified 6 SNPs, two 1 bp indels (positions 157 and 334 bp) and a 7 bp indel (position 351 bp). Although, 18 animals were included in this analysis, only haplotypes identified in individuals caring two copies of the same haplotype were taken into account because the phase could not be established in heterozygous individuals. In all sequenced animals, alleles of indels at 334 and 351 bp and SNP-26749 were in complete linkage with each other forming two haplotypes (haplotypes 1 and 2). An indel at 157 bp was identified only in conventional farm-bred fox population. A 1 bp insertion at 157 bp observed in this population was identified in haplotype 2, forming novel haplotype (haplotype 3) in conventional farm-bred fox population.

Because this genomic region contains an indel and is located close to the SNP S1_1977727423, it was selected for genotyping of additional samples from three fox populations. Using fluorescently labeled primers (Table S3) which amplified a region including all three indels, a set of foxes comprising tame (46), aggressive (45), and conventional farm-bred foxes (92) was genotyped. Two amplicons were identified in the tame and aggressive samples, 372 and 378, and an additional amplicon, 373 bp, was identified in 15 samples from conventional farm-bred foxes. Amplicon 378 corresponded to haplotype 1 while amplicons 372 and 373 corresponed to haplotypes 2 and 3, respectively. In the Fisher exact test the amplicons 372 and 373 were analyzed together due to complete linkage of an insertion at 157 bp with haplotype 2.

	Indel	SNP26749	Indel	Indel	Tame	Aggr	Conv	Total
	157 bp	263 bp	334 bp	351 bp				
Haplotype								
Hapl#1	$(T)_7$	C	$(C)_1$	CCCACCT	0	5	2	7
Hapl#2	$(T)_7$	T	$(C)_2$	-	4	1	3	7
Hapl#3	$(T)_8$	T	$(C)_2$	-	0	0	4	4
