# Supplementary material for: Genotyping-By-Sequencing (GBS) Detects Genetic Structure and Confirms Behavioral QTL in Tame and Aggressive Foxes (Vulpes vulpes)
Source: PLoS One. 2015 Jun 10;10(6):e0127013. doi: 10.1371/journal.pone.0127013 (PMC4465646; doi:10.1371/journal.pone.0127013)
Supplement: S1 Table — (PDF) [file pone.0127013.s006.pdf]

Table S1. Adapters used for construction of *Eco*T22I library. The adapters comprised a set of 48 different barcode-containing adapters (A and B) and a "common" adapter.

**Barcode-containing adapters:**					
A	5′-ACACTCTTTCCCTACACGACGCTCTTCCGATCTxxxxTGCA				
B	5′-yyyyAGATCGGAAGAGCGTCGTGTAGGGAAAGAGTGT				
**Barcodes "xxxx":**					
TGACGCCA	CAGATA	GAAGTG	TAGCGGAT	TATTCGCAT	ATAGAT
CCGAACA	GGAAGACAT	GGCTTA	AACGCACATT	GAGCGACAT	CCTTGCCATT
GGTATA	TCTTGG	GGTGT	GGATA	CTAAGCA	ATTAT
GCGCTCA	ACTGCGAT	TTCGTT	ATATAA	TGGCAACAGA	CTCGTCG
GCCTACCT	CACCA	AATTAG	GGAACGA	ACAACT	ACTGCT
CGTGGACAGT	TGGCACAGA	TGCTT	GCAAGCCAT	CGCACCAATT	CTCGCGG
AACTGG	ATGAGCAA	CTTGA	GCGTCCT	ACCAGGA	CCACTCA
TCACGGAAG	TATCA	TAGCCAA	ATATCGCCA	CTCTA	GGTGCACATT
**Common adapters:**					
C	5′-AGATCGGAAGAGCGGTTCAGCAGGAATGCCGAG				
D	5′-CTCGGCATTCCTGCTGAACCGCTCTTCCGATCTTGCA				
