# Supplementary material for: Genotyping-By-Sequencing (GBS) Detects Genetic Structure and Confirms Behavioral QTL in Tame and Aggressive Foxes (Vulpes vulpes)
Source: PLoS One. 2015 Jun 10;10(6):e0127013. doi: 10.1371/journal.pone.0127013 (PMC4465646; doi:10.1371/journal.pone.0127013)
Supplement: S2 Table — (PDF) [file pone.0127013.s007.pdf]

Table S2. Bins used for calculation of average r^2 between SNPs located on the same chromosome.

**Bin length**	**Number of SNP pairs in Tame population**	**Percent of SNP pairs with r^2=1 in Tame population**	**Number of SNP pairs in Aggressive population**	**Percent of SNP pairs with r^2=1 in Aggressive population**
1-1000 bp	4,723	66.27%	5,294	62.56%
1 kb-5 kb	136	31.62%	170	20.59%
5 kb-10 kb	185	16.22%	219	21.46%
10 kb-20 kb	471	12.10%	502	7.37%
20 kb-50 kb	1,004	10.26%	1,184	6.17%
50 kb-100 kb	1,686	6.52%	1,998	3.60%
100 kb-500 kb	11,752	5.50%	14,451	2.15%
500 kb-1 Mb	14,407	2.91%	17,654	1.43%
1 Mb-5 Mb	69,717	1.48%	120,932	0.69%
5 Mb-10 Mb	104,237	0.67%	131,928	0.21%
10 Mb-20 Mb	176,447	0.40%	221,294	0.12%
20 Mb-50 Mb	355,304	0.25%	441,773	0.11%
50 Mb-100 Mb	430,446	0.16%	537,138	0.06%
100 Mb-210Mb	267,573	0.10%	328,622	0.03%
