# Supplementary material for: Genotyping-By-Sequencing (GBS) Detects Genetic Structure and Confirms Behavioral QTL in Tame and Aggressive Foxes (Vulpes vulpes)
Source: PLoS One. 2015 Jun 10;10(6):e0127013. doi: 10.1371/journal.pone.0127013 (PMC4465646; doi:10.1371/journal.pone.0127013)
Supplement: S3 Table — (PDF) [file pone.0127013.s008.pdf]

Table S3. Florescent primers used for genotyping S1_1977727423 associated indels and SSRs markers on VVU3.

Marker	Forward	Reverse	Dye	Amplicon size
26749b	CTGGTCTGGGCTCCTTGTCTCA	TCGGGAGAACAGAGGGTAAGGA	NED	372-378
VV0683	CCGGCCACCAGATACAATTT	AGCTGCTCAGAGCCACACTG	VIC	363-390
CM6.72b	GCTGTCCAAAATGCAGACAA	TCTCAGTAGCCCGAGGAAGA	6-FAM	151-172
CM6.75	TTGAAGCCATCTGAAAGCAG	CAGATGGCAAGACCTCTTCA	VIC	178-195
