# Supplementary material for: Genotyping-By-Sequencing (GBS) Detects Genetic Structure and Confirms Behavioral QTL in Tame and Aggressive Foxes (Vulpes vulpes)
Source: PLoS One. 2015 Jun 10;10(6):e0127013. doi: 10.1371/journal.pone.0127013 (PMC4465646; doi:10.1371/journal.pone.0127013)
Supplement: S5 Table — (PDF) [file pone.0127013.s010.pdf]

Table S5. SNP evaluation statistics.

(A) Analysis of heterozygosity of SNPs whose positions were assigned to the X chromosome outside of pseudoautosomal region (PAR). SNPs assigned to the PAR (0-6.65Mb on dog X chromosome) were not included in this analysis. The filtered set (48,294 SNPs) included 1,065 of SNPs located on X chromosome outside of PAR. The stringently filtered SNP set (8,437 SNPs) included 105 SNPs located on X chromosome outside of PAR.

SNP set	Population	Average percent of heterozygous males per SNP	Average percent of heterozygous females per SNP
Filtered set	Tame	5.4%	12.4%
	Aggressive	5.4%	13.9%
Stringently filtered set	Tame	1.2%	25.2%
	Aggressive	1.0%	29.4%

(B) Evaluation of the SNP call reproducibility in duplicated samples.

SNP set	Percent of SNPs for which genotypes of replicates were in perfect concordance	The average read depth for the discordant SNPs	The average read depth for the concordant SNPs
Filtered set	66.5-73.8%	14.0 ± 25.4	34.0 ± 41.6
Stringently filtered set	97.9-99.2%	55.3 ± 39.8	76.1 ± 38.2
