# Supplementary material for: Genotyping-By-Sequencing (GBS) Detects Genetic Structure and Confirms Behavioral QTL in Tame and Aggressive Foxes (Vulpes vulpes)
Source: PLoS One. 2015 Jun 10;10(6):e0127013. doi: 10.1371/journal.pone.0127013 (PMC4465646; doi:10.1371/journal.pone.0127013)
Supplement: S6 Table — (PDF) [file pone.0127013.s011.pdf]

Table S6. Genomic regions highlighted in the fixation index (F_{ST}) analysis. Windows with $F_{ST} \geq 0.5$ are listed. The fox chromosomes with more than one significant window and syntenic regions in the dog genome are shaded in gray. Windows which include SNPs reported in Table 2 are highlighted in yellow.

CFA	Start position in the Dog genome (bp)	End position in the Dog genome (bp)	VVU	Start position in the Fox genome (bp)	End position in the Fox genome (bp)	Number of SNPs in the window	Weighted F_{ST}
1	29,500,001	30,500,000	1	92,178,785	93,178,785	5	0.56
6	66,000,001	67,000,000	3	138,935,427	139,935,427	12	0.54
6	68,500,001	69,500,000	3	141,435,427	142,435,427	9	0.59
8	32,500,001	33,500,000	6	93,939,935	94,939,935	12	0.60
8	67,000,001	68,000,000	6	128,439,935	129,439,935	25	0.51
27	28,000,001	29,000,000	8	28,000,001	29,000,001	18	0.63
27	29,000,001	30,000,000	8	29,000,001	30,000,001	19	0.51
27	32,000,001	33,000,000	8	32,000,001	33,000,001	27	0.54
27	32,500,001	33,500,000	8	32,500,001	33,500,001	29	0.64
27	33,000,001	34,000,000	8	33,000,001	34,000,001	15	0.61
27	35,000,001	36,000,000	8	35,000,001	36,000,001	6	0.56
27	35,500,001	36,500,000	8	35,500,001	36,500,001	15	0.65
27	36,000,001	37,000,000	8	36,000,001	37,000,001	13	0.61
27	44,500,001	45,500,000	8	44,500,001	45,500,001	4	0.62
27	45,000,001	46,000,000	8	45,000,001	46,000,001	4	0.62
17	57,000,001	58,000,000	8	52,165,769	53,165,769	3	0.84
17	56,500,001	57,500,000	8	52,665,769	53,665,769	16	0.54
17	51,500,001	52,500,000	8	57,665,769	58,665,769	7	0.73
17	51,000,001	52,000,000	8	58,165,769	59,165,769	3	0.78
17	48,000,001	49,000,000	8	61,165,769	62,165,769	8	0.52
17	38,500,001	39,500,000	8	70,665,769	71,665,769	11	0.60
17	38,000,001	39,000,000	8	71,165,769	72,165,769	6	0.56
17	36,000,001	37,000,000	8	73,165,769	74,165,769	4	0.51
7	41,500,001	42,500,000	13	122,752,749	123,752,749	1	0.51
3	43,000,001	44,000,000	14	90,698,780	91,698,780	23	0.66
3	43,500,001	44,500,000	14	91,198,780	92,198,780	16	0.66
X	104,500,001	105,500,000	X	104,500,001	105,500,001	8	0.50
X	114,000,001	115,000,000	X	114,000,001	115,000,001	14	0.52
